# EXAMINING LONGITUDINAL HEALTHCARE UTILIZATION, OUTCOMES, AND SATISFACTION IN PRE-FRAIL OLDER ADULTS

**DOI:** 10.1093/geroni/igz038.2541

**Published:** 2019-11-08

**Authors:** Ivana A Vaughn, Nicole M Marlow, Kalyani Sonawane, Roger B Fillingim, Rebecca J Beyth

**Affiliations:** 1 The New York Academy of Medicine, New York, New York, United States; 2 University of Florida College of Public Health and Health Professions, Gainesville, Florida, United States; 3 The University of Texas Health Science Center at Houston School of Public Health, Houston, Texas, United States; 4 Pain Research and Intervention Center of Excellence, University of Florida, Gainesville, United States; 5 Geriatric Research Education and Clinical Center, North Florida South Georgia Veterans Health System,, Gainesville, Florida, United States

## Abstract

Frailty is a medical syndrome occurring in nearly 60% community dwelling older adults and could have insidious origins in middle-adulthood that manifest predominantly as decline and multi-morbidity. Identification of pre-frail states in adults could potentially reduce its impact in late-life. The study used data from the Health and Retirement Study (HRS) to: 1) compare long-term healthcare utilization between pre-frail and non-frail patients, 2) compare risk of pain progression, functional decline, and mortality between pre-frail and non-frail patients, and (3) compare satisfaction with healthcare, self-perceptions of aging and satisfaction with life between pre-frail and non-frail patients. The primary predictor, pre-frailty phenotype, was based on the Paulson-Lichtenberg Frailty Index (PLFI), a validated HRS-version of the Fried criteria. Additional covariates included sociodemographics, comorbidities, smoking status, sleep quality, health status, and body mass index. Statistical analyses consisted of descriptive statistics, univariate analysis, negative binomial regression with log-link function, logistic regression, generalized linear modeling and Cox regression. Pre-frailty status increased the incidence of hospitalizations (IRR: 1.23; 95%CI: 1.18-1.28) and doctor’s visits (IRR: 1.18; 95%CI: 0.15-1.22); increased the risk of pain progression (HR: 1.61, 95%CI: 1.53-1.69), functional decline (HR: 1.77, 95%CI: 1.67-1.87), and all-cause mortality (HR: 1.09, 95%CI:1.03-1.16); lowered odds of healthcare satisfaction (OR: 0.79, 95%CI: 0.62-0.99) and lowered satisfaction with aging ((β: -0.23 95%CI: -0.36- (-0.10)) and satisfaction with life (β: -0.27 95%CI: -0.44- (-0.11)). Frailty syndrome is highly prevalent and having a better understanding of its influence on health outcomes at intermediate pre-frail states could provide insight into reducing manifestations in later life.

